# Clinical effectiveness of oral antiviral treatment for non-hospitalized high-risk patients with COVID-19 during Omicron JN.1 subvariant wave: a US-based propensity-matched cohort study

**DOI:** 10.1186/s41479-025-00168-w

**Published:** 2025-05-25

**Authors:** Wan-Hsuan Hsu, Bo-Wen Shiau, Po-Yu Huang, Ya-Wen Tsai, Jheng-Yan Wu, Ting-Hui Liu, Min-Hsiang Chuang, Shu-Farn Tey, Lun-Wu Hung, Chih-Cheng Lai

**Affiliations:** 1 Department of Internal Medicine, Chi Mei Medical Center, Tainan, Taiwan; 2 Preventitve Medicine Division, Chi Mei Medical Center, Tainan, Taiwan; 3 Department of Medical Laboratory Sciences and Biotechnology, Fooyin University, Kaohsiung, Taiwan; 4 Department of Nutrition, Chi Mei Medical Center, Tainan, Taiwan; 5 Department of Public Health, College of Medicine, National Cheng Kung University, Tainan, Taiwan; 6 Department of Psychiatry, Chi Mei Medical Center, Tainan, Taiwan; 7 Division of Cardiovascular Surgery, Department of Surgery, Chi Mei Medical Center, Chiali, Tainan, Taiwan; 8 Department of Intensive Care Medicine, Chi Mei Medical Center, Tainan, Taiwan; 9 School of Medicine, College of Medicine, National Sun Yat-Sen University, Kaohsiung, Taiwan

**Keywords:** JN.1, COVID-19, SARS-CoV-2, Antiviral

## Abstract

**Background:**

This real-world study aimed to assess the effectiveness of novel oral antiviral agents in managing COVID-19 among high-risk patients during the Omicron JN.1 subvariant wave.

**Methods:**

Data from the TriNetX US network were analyzed using a multi-institutional propensity score matching (PSM) analysis. High-risk non-hospitalized adults with COVID-19 were included, and patients receiving oral antiviral agents (study group) were compared to those not receiving antiviral agents (control group). Primary outcomes included all-cause emergency department (ED) visits, hospitalizations, or death within 30 days.

**Results:**

Among 67,495 high-risk patients identified, 17,852 received oral antiviral agents (study group) and 49,643 did not (control group). After PSM, two matched cohorts of 17,847 patients each were established. The study group receiving antiviral agents exhibited a significantly lower risk of primary composite outcome during the 30-day follow-up period compared to the control group (HR, 0.77; 95% CI, 0.72–0.84). Regarding the secondary outcomes, the study group consistently exhibited a significantly lower risk of all-cause ED visits (4.2% vs. 5.4%; HR, 0.78; 95% CI, 0.71–0.86), hospitalization (2.8% vs. 3.3%; HR, 0.86; 95% CI, 0.77–0.97), and mortality (0.1% vs. 0.3%; HR, 0.17; 95% CI, 0.08–0.35) than the control group. Subgroup analyses showed consistent benefits across various demographic and clinical characteristics, except in individuals with booster vaccination.

**Conclusions:**

Oral antiviral agents significantly reduced the risk of adverse outcomes among high-risk COVID-19 patients during the Omicron JN.1 subvariant wave. These findings support the potential benefits of oral antiviral therapy in treating COVID-19, particularly in high-risk populations.

**Supplementary Information:**

The online version contains supplementary material available at 10.1186/s41479-025-00168-w.

## Introduction

In late 2019, the first outbreak of SARS-CoV-2 infection occurred in China [1]. Subsequently, COVID-19 rapidly spread worldwide and was declared a pandemic in March 2020 [2]. By February 2025, there had been over 777 million cases of COVID-19, resulting in more than 7 million deaths [3]. Global efforts to develop effective vaccines and antiviral treatments against SARS-CoV-2 gradually brought COVID-19 under control, leading the World Health Organization to declare an end to its status as a public health emergency on May 5, 2023. However, SARS-CoV-2 persisted and continued to evolve, underscoring the ongoing global threat posed by COVID-19. Given the potential variability in characteristics among SARS-CoV-2 variants, it is imperative to gather reliable evidence early on to ascertain whether new variants are more transmissible, virulent, or resistant to existing COVID-19 vaccines and treatments [4–7].

Recently, the emergence of JN.1, which evolved from the BA.2.86 sub-variant, has been observed since late 2023, rapidly spreading across various countries and becoming the dominant strain in certain regions by early 2024 [8–12]. The mutations present in JN.1 have the potential to augment its infectivity and evade immune responses [9, 13]. Despite the heightened global concern due to its rapid dissemination, the implications of JN.1 mutations on the course of the pandemic and the efficacy of current vaccines and treatments remain uncertain [14].

While many infections attributed to SARS-CoV-2 manifest as mild-to-moderate, a notable subset of COVID-19 patients would develop severe-to-critical illness, especially among high-risk individuals [15]. Hence, it is imperative to establish effective treatments for high-risk patients to mitigate the progression of COVID-19. Hence, it is imperative to establish effective treatments for high-risk patients to mitigate the progression of COVID-19. Despite many studies having demonstrated the effectiveness of nirmatrelvir-ritonavir (NMV-r) and molnupiravir (MOV) in the treatment of patients infected by SARS-CoV-2 Omicron variants, including XBB subvariants [16–20], the effect of oral antiviral agents against the new subvariant—JN.1 sub-variant was unknown. Therefore, this first real-world study was conducted using a multi-institutional propensity score match (PSM) analysis to establish evidence regarding the effectiveness of novel oral antiviral agents in the treatment of non-hospitalized high-risk patients with COVID-19 during the Omicron JN.1 subvariant wave.

## Method

### Data source

TriNetX serves as a global health-collaborative clinical-research platform, offering researchers access to real-time electronic medical data sourced from over 120 healthcare organizations (HCOs) across 19 countries in North and South America, Asia–Pacific, Europe, the Middle East, and Africa [21]. This retrospective cohort study focused on data from the US Collaborative Network due to epidemiological considerations regarding the spread of JN.1 subvariants referencing Centers for Disease Control and Prevention (CDC)’s Variants and Genomic Surveillance for SARS-CoV-2 [22]. Comprehensive patients’ data including diagnostic records, procedural histories, medication details, laboratory results, and genomic information can be directly extracted from participating institutions' electronic health record (EHR) systems, with stringent measures in place to ensure data integrity and standardization. De-identification procedures are rigorously implemented, and the database provides aggregated counts and statistical summaries for relevant variables [21]. The requirement for written informed consent was waived because all the obtained data were in aggregate deidentified form, and this study was approved by the Institutional Review Board ofC Chi Mei Medical Center (approval number 11202–002).

### Study design and patient selection

This retrospective cohort study was in alignment with a Target Trial Emulation [23] evaluating the effect of novel oral antiviral agents on short-risk of emergency department (ED) visits, hospitalization, and death among non-hospitalized high-risk adults with COVID-19. First, we identified a cohort of high-risk patients with COVID-19 on May 6, 2024. The inclusion criteria were a) having at least three medical visits with HCOs from February 1, 2024, to April 30, 2024; b) aged > 18 years; and c) presence of risk factor of severe COVID-19. These risk factors included any of the following conditions: cardiovascular condition, cerebrovascular disease, chronic liver disease, chronic lower lung disease, diabetes mellitus (DM), hypertensive disease, immune disorder, overweight/obesity, psychiatric disease, and malignancy [24]. These conditions were defined using the International Classification of Diseases, Tenth Revision, Clinical Modification (ICD-10-CM) codes. Second, we identified patients with a diagnosis of COVID-19 or a recorded positive PCR test for COVID-19 based on the ICD-10-CM code U07.1 (“COVID-19”) or a positive SARS-CoV-2 and related RNA laboratory test result (TNX: LAB:9088), as previously described [25, 26]. Third, we excluded patients receiving other recommended treatments for non-hospitalized patients with COVID-19, including convalescent plasma, neutralizing monoclonal antibodies (bebtelovimab, cilgavimab, and tixagevimab), and remdesivir, as well as patients who were hospitalized between 2 days before and 5 days after COVID-19 diagnosis. Finally, the patient population was stratified into two cohorts: those receiving NMV-r or MOV (the study group) and those not receiving novel antiviral agents (the control group) (Supplementary Table 1). For all included patients, the following information including demographic characteristics (i.e., age, sex, and ethnicity), lifestyle, and underlying comorbidities were collected (Supplementary Table 2) [17]. Index date was defined as the first of receiving antiviral agents for the study group and the first date of COVID-19 diagnosis for the control group.

### Outcome

The main composite outcome was all-cause ED visit, hospitalization, or death during the 30-day follow-up. Secondary outcomes were individual components of the primary outcomes: all-cause ED visits, hospitalization, and death. These outcomes were identified according to curated demographic coding of the TriNetX platform, as described previously [27] (Supplementary Table 3).

### Statistical analysis

Continuous variables were described using mean ± standard deviation, and categorical variables using frequency (n) and percentage (%). Comparisons between the two cohorts were made using independent t-tests for continuous variables and chi-square tests for categorical variables. To adjust for baseline differences, propensity score matching at a 1:1 ratio was conducted using the built-in function of the TriNetX network, which incorporates a greedy nearest-neighbor algorithm with a caliper setting of 0.1 pooled standard deviations. Characteristics were considered well-balanced if the standardized mean difference was less than 0.1 [28].

Cox proportional hazard regression was used to calculate the hazard ratios (HRs) with 95% confidence intervals (CIs). Kaplan–Meier curves were used to determine cumulative probability. Statistical significance was set at *p* < 0.05. Subgroup analysis by age, sex, underlying disease, vaccine status (with or without booster dose), the episode of SARS-CoV-2 infection (primary or reinfection), the antiviral treatment (NMV-r and MOV) was conducted (Supplementary Table 4). Statistical analyses were completed with the use of the TriNetX online platform using Stata for statistical computing.

## Results

### Study subjects

**Fig. 1:**
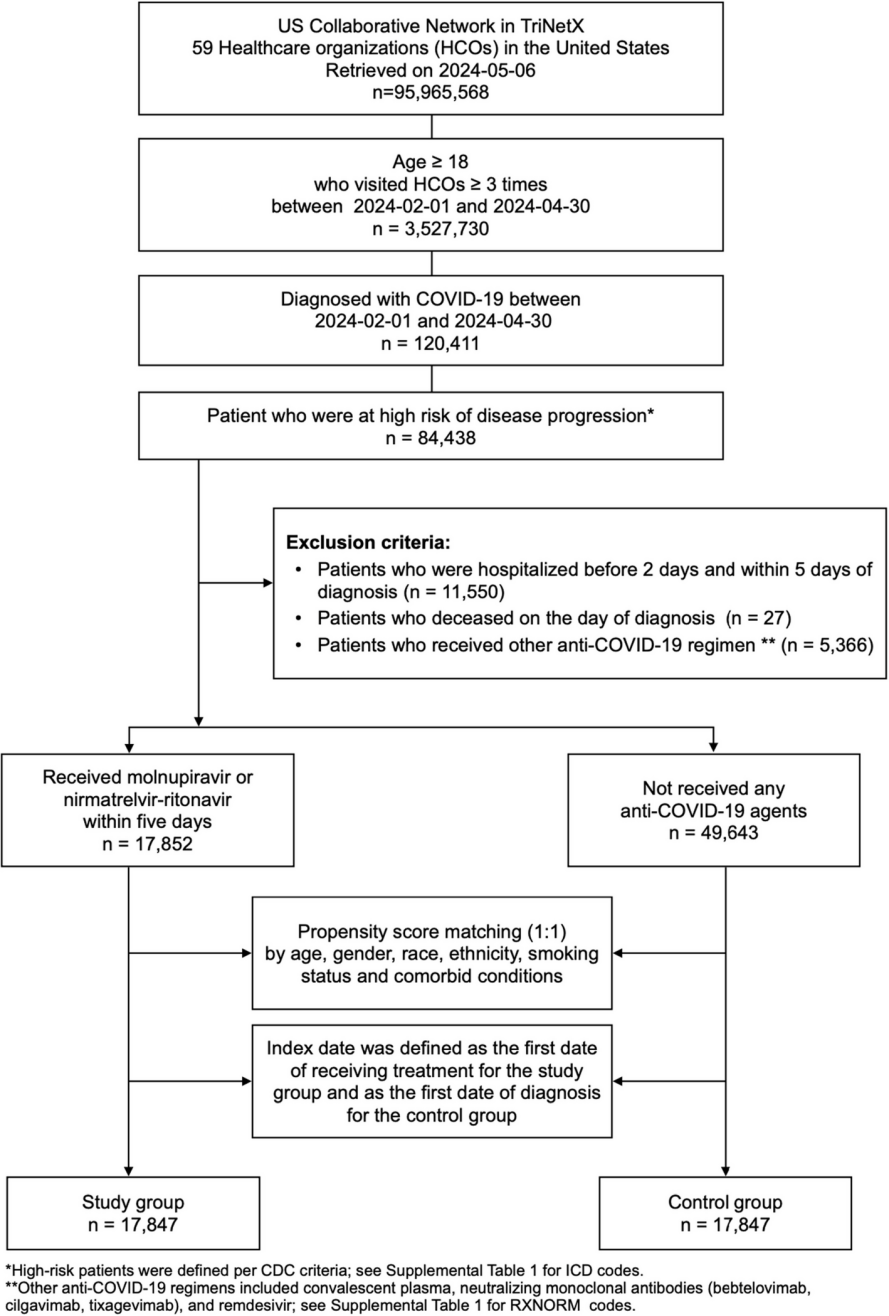
Flowchart of patient selection and cohort construction

Based on the initial database search, a total of 84,438 high-risk patients with COVID-19 were identified during the study period. After excluding those requiring initial hospitalization, with death on the index date, and receiving other anit-COVID-19 medications, 17,852 patients received oral antiviral agents (study group), while 49,643 patients did not receive antiviral agents (control group). Table 1 presents the baseline characteristics of the patients included in the analysis before and after PSM. Before match, the study group exhibited older age demographics compared to the control group, with a mean age of 61.8 ± 15.6 years versus 57.9 ± 18.3 years, respectively. Furthermore, there was a lower proportion of Black or African American individuals in the study group compared to the control group (7.8% versus 13.7%). Additionally, there was a higher prevalence of pulmonary heart disease and schizophrenia in the control group compared to the study group. Following PSM, two matched cohorts of 17,847 patients each were established, resulting in a total of 35,694 patients included in the analysis. The absolute standardized mean differences between the study and control groups were all < 0.1, indicating successful matching (Fig. 1).

**Table 1:** Baseline characteristics in the study and control groups before and after propensity score matching (PSM)

	**Before PSM**			**After PSM**		
	Study group^a^ (*N* = 17,852)	Control group^b^ (*N* = 49,643)	Std diff.^c^	Study group (*N* = 17,847)	Control group (*N* = 17,847)	Std diff
**Demographics**						
Age at Index (mean ± SD)	61.8 ± 15.6	57.9 ± 18.3	0.227	61.8 ± 15.6	62.0 ± 17.1	0.014
* Sex (%)*						
Female	10,128 (56.7)	30,384 (61.2)	0.091	10,128 (56.7)	10,150 (56.9)	0.002
Male	6,139 (34.4)	16,500 (33.2)	0.024	6,138 (34.4)	6,189 (34.7)	0.006
* Race (%)*						
White	12,967 (72.6)	34,574 (69.6)	0.066	12,966 (72.7)	13,083 (73.3)	0.015
Black or African American	1,389 (7.8)	6,808 (13.7)	0.192	1,389 (7.8)	1,352 (7.6)	0.008
Asian	605 (3.4)	1,535 (3.1)	0.017	605 (3.4)	580 (3.2)	0.008
American Indian or Alaska Native	43 (0.2)	164 (0.3)	0.017	43 (0.2)	41 (0.2)	0.002
Native Hawaiian or Other Pacific Islander	47 (0.3)	168 (0.3)	0.014	47 (0.3)	50 (0.3)	0.003
Other Race	510 (2.9)	1,300 (2.6)	0.015	510 (2.9)	491 (2.8)	0.006
Unknown Race	2,291 (12.8)	5,094 (10.3)	0.081	2,287 (12.8)	2,250 (12.6)	0.006
* Ethnicity (%)*						
Not Hispanic or Latino	14,607 (81.8)	40,930 (82.4)	0.016	14,606 (81.8)	14,681 (82.3)	0.011
Hispanic or Latino	1,136 (6.4)	4,151 (8.4)	0.077	1,136 (6.4)	1,111 (6.2)	0.006
Unknown Ethnicity	2,109 (11.8)	4,562 (9.2)	0.086	2,105 (11.8)	2,055 (11.5)	0.009
**Smoking status (%)**						
Nicotine dependence	2,159 (12.1)	8,322 (16.8)	0.133	2,159 (12.1)	2,169 (12.2)	0.002
Personal history of nicotine dependence	3,340 (18.7)	11,944 (24.1)	0.131	3,340 (18.7)	3,305 (18.5)	0.005
**Comorbid conditions (%)**						
* Cardiovascular diseases*						
Hypertensive diseases	11,711 (65.6)	30,662 (61.8)	0.08	11,707 (65.6)	11,717 (65.7)	0.001
Ischemic heart diseases	3,876 (21.7)	11,521 (23.2)	0.036	3,876 (21.7)	3,954 (22.2)	0.011
Pulmonary heart diseases	966 (5.4)	4,193 (8.4)	0.12	966 (5.4)	964 (5.4)	< 0.001
Cerebrovascular diseases	2,188 (12.3)	7,039 (14.2)	0.057	2,188 (12.3)	2,172 (12.2)	0.003
Neoplasms	7,758 (43.5)	20,957 (42.2)	0.025	7,753 (43.4)	7,752 (43.4)	< 0.001
Schizophrenia	290 (1.6)	1,577 (3.2)	0.102	290 (1.6)	291 (1.6)	< 0.001
Mood disorders	5,963 (33.4)	18,557 (37.4)	0.083	5,961 (33.4)	5,911 (33.1)	0.006
Anxiety disorders	7,679 (43.0)	22,834 (46.0)	0.06	7,677 (43.0)	7,548 (42.3)	0.015
Overweight and obesity	6,718 (37.6)	18,641 (37.6)	0.002	6,716 (37.6)	6,653 (37.3)	0.007
Diabetes mellitus	5,288 (29.6)	14,574 (29.4)	0.006	5,284 (29.6)	5,274 (29.6)	0.001
Immune disorders	964 (5.4)	3,758 (7.6)	0.088	963 (5.4)	934 (5.2)	0.007
Human immunodeficiency virus disease	275 (1.5)	716 (1.4)	0.008	274 (1.5)	246 (1.4)	0.013
Chronic kidney disease	2,837 (15.9)	8,475 (17.1)	0.032	2,836 (15.9)	2,813 (15.8)	0.004
Chronic lower respiratory diseases	5,926 (33.2)	17,403 (35.1)	0.039	5,924 (33.2)	5,971 (33.5)	0.006
Cystic fibrosis	19 (0.1)	111 (0.2)	0.029	19 (0.1)	17 (0.1)	0.004
Chronic liver disease	2,493 (14.0)	7,667 (15.4)	0.042	2,490 (14.0)	2,449 (13.7)	0.007

^a^Study group: with nirmatrelvir-ritonavir or molnupiravir use

^b^Control group: without any anti-COVID-19 agents use

^c^Std diff.: standardized difference. A std diff. of less than 0.1 was considered evidence of good balance

### Primary outcome

**Fig. 2:**
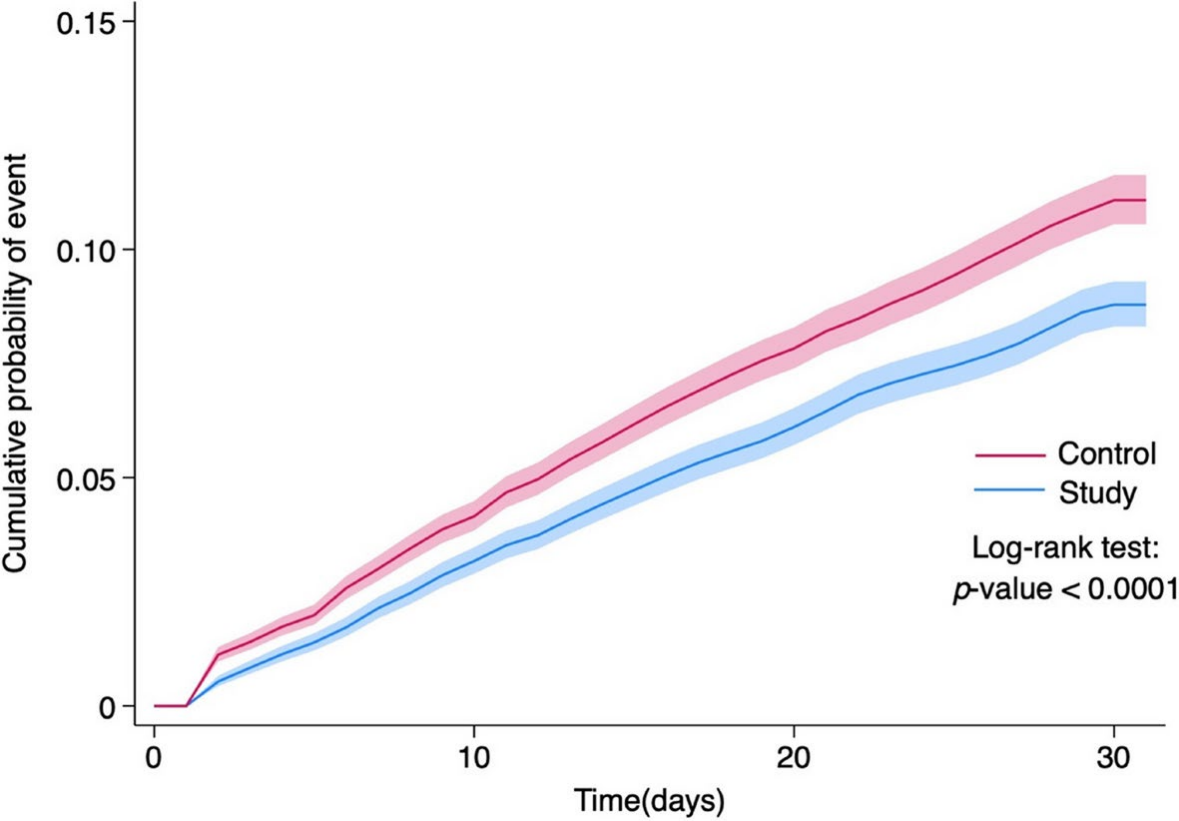
Cumulative probability of all-cause ED visit, hospitalization or mortality during the follow-up 30 days

During the 30-day follow-up period, 6.3% (*n* = 1130) of the study group and 8.2% (*n* = 1468) of the control group developed the primary composite outcomes of all-cause ED visit, hospitalization, or mortality. Compared to the control group, the study group exhibited a significantly lower risk of primary outcomes (HR, 0.77; 95% CI, 0.72–0.84) (Table 2). Additionally, Kaplan–Meier curves of the probability of time-to-event revealed a significantly lower risk of all-cause ED visit, hospitalization, or mortality in the oral antiviral group compared to the control group (*p* < 0.0001, log-rank test) (Fig. 2).

**Table 2:** The hazard ratio and incidence for comparing matched study and control groups for the primary composite outcome and its constituents

Outcomes	No. (%) of patients with outcome		HR (95% CI)^c^	Log-rank test *p*-value
	**Study group**^**a**^	**Control group**^**b**^		
**Primary outcome**				
All-cause ED visit^d^, hospitalization or mortality	1,130 (6.3)	1,468 (8.2)	0.77 (0.72, 0.84)	< 0.0001
**Secondary outcomes**				
All-cause ED visit	743 (4.2)	961 (5.4)	0.78 (0.71, 0.86)	< 0.0001
All-cause hospitalization	507 (2.8)	596 (3.3)	0.86 (0.77, 0.97)	0.0136
All-cause mortality	10 (0.1)	49 (0.3)	0.17 (0.08, 0.35)	< 0.0001

^a^Study group: with nirmatrelvir-ritonavir or molnupiravir use

^b^Control group: without any anti-COVID-19 agents use

^c^HR (95% CI): hazard ratio (95% confidence interval)

^d^ED visit: emergency department visit

### Secondary outcomes

**Fig. 3:**
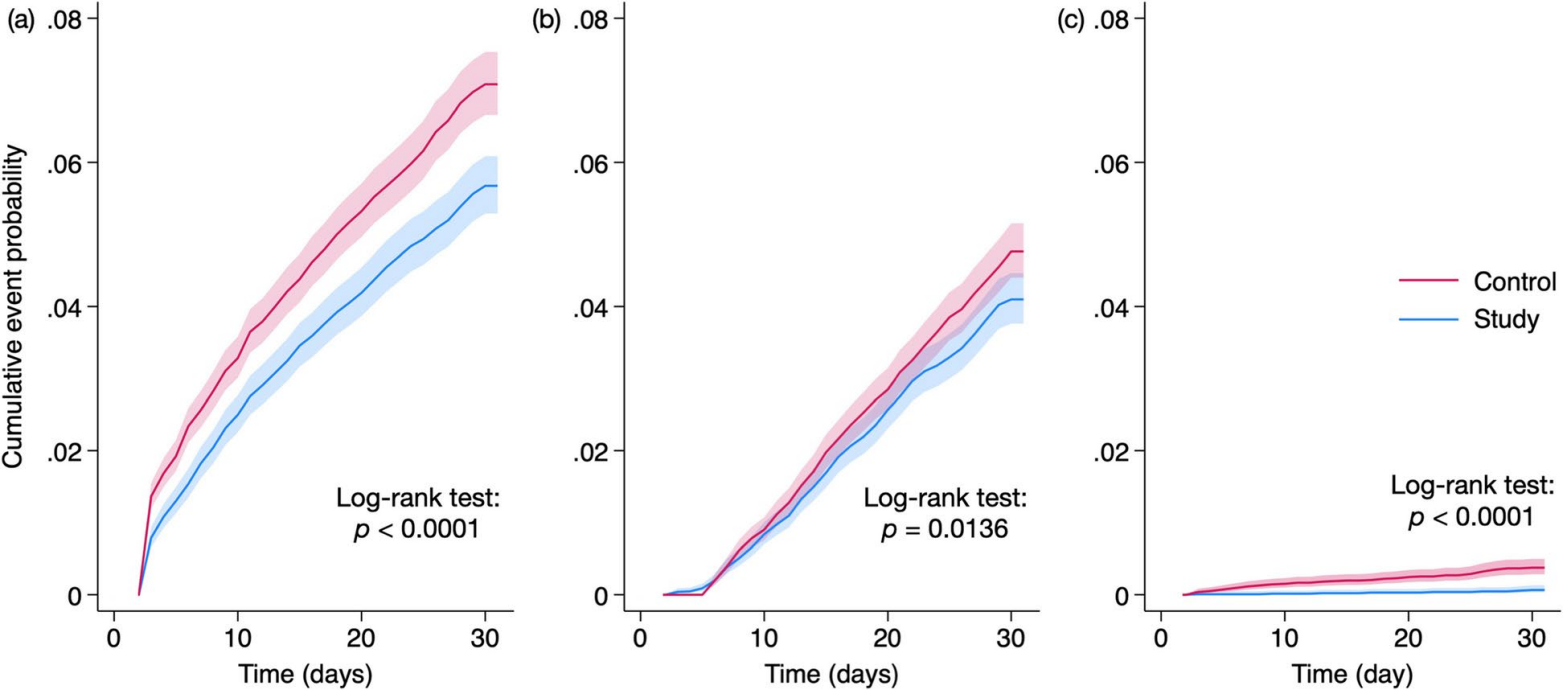
Cumulative probability of (**a**) all-cause ED visit, (**b**) all-cause hospitalization, and (**c**) all-cause mortality during the follow-up 30 days

Regarding the secondary outcomes, the study group consistently exhibited a significantly lower risk of all-cause ED visits (4.2% vs. 5.4%; HR, 0.78; 95% CI, 0.71–0.86), hospitalization (2.8% vs. 3.3%; HR, 0.86; 95% CI, 0.77–0.97), and mortality (0.1% vs. 0.3%; HR, 0.17; 95% CI, 0.08–0.35) than the control group. Additionally, Kaplan–Meier curves of the probability of time-to-event showed a significantly lower risk of all-cause ED visit, hospitalization, or mortality in the study group compared to the control group (all *p* < 0.05, log-rank test) (Fig. 3).

### Subgroup analyses

The significantly lower risk of composite outcomes—all-cause ED visit, hospitalization, or mortality in the study group was consistently observed across almost all subgroups, except individual with booster vaccine (Fig. 3). This included individuals aged < 65 years (HR, 0.75 [0.67–083]) and those aged ≥ 65 years (HR, 0.81 [0.72–0.90]), as well as females (HR, 0.77 [0.70–0.85]) and males (HR, 0.84 [0.74–0.96]). Moreover, patients with various comorbidities such as cardiovascular disease (HR, 0.76 [0.70–0.84]), cerebrovascular disease (HR, 0.77 [0.61–0.97]), chronic liver disease (HR, 0.68 [0.54–0.86]), chronic lower respiratory diseases (HR, 0.80 [0.70–0.91]), chronic kidney disease (HR, 0.69 [0.57–0.83]), and DM (HR, 0.79 [0.69–0.91]), as well as those with overweight and obesity (HR, 0.75 [0.65–0.87]), all showed a reduced risk of primary outcomes when treated with oral antiviral agents.

**Fig. 4:**
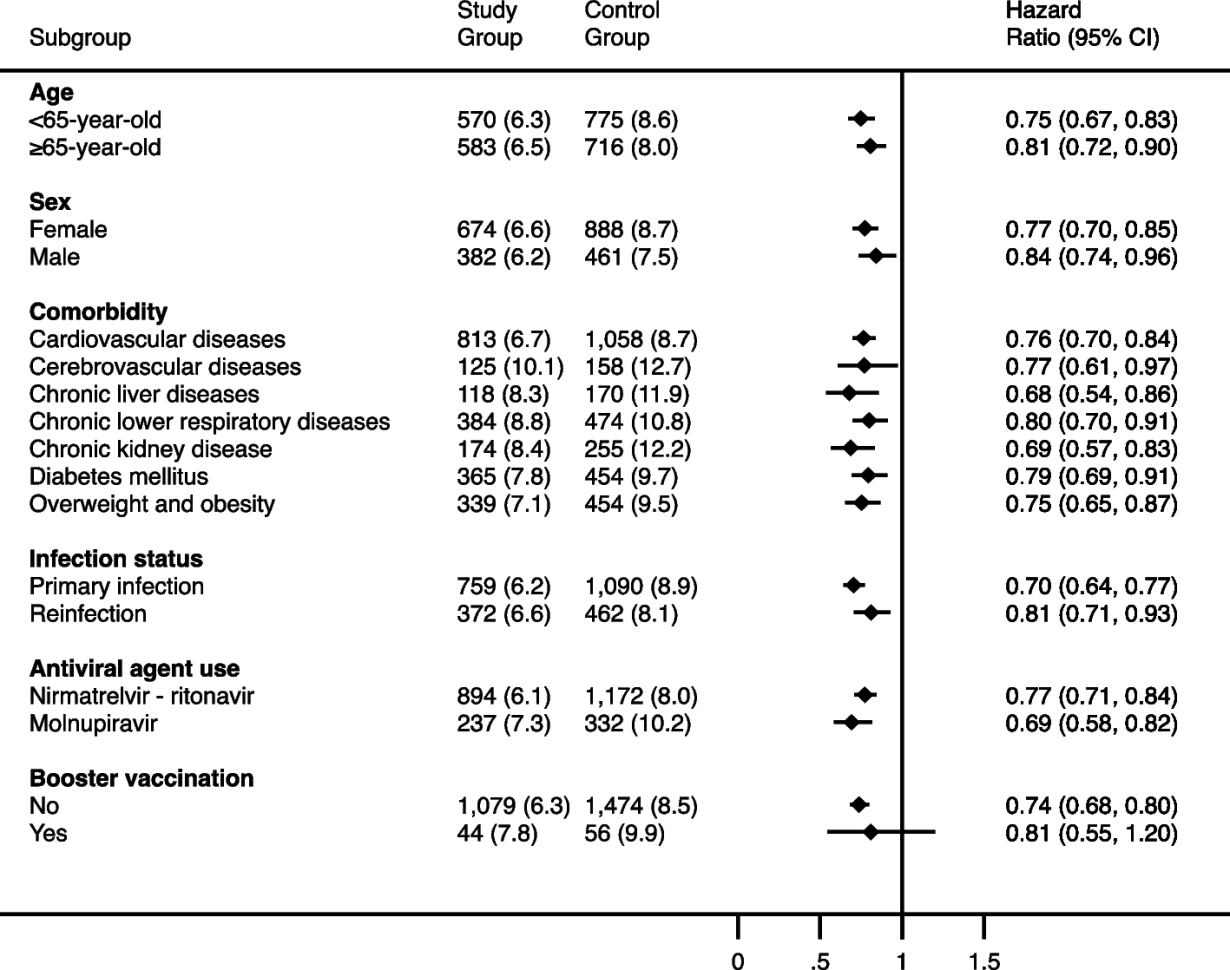
Hazard ratio and percentage of primary outcome in the subgroup of the study group compared with the control group

Similarly, the reduced risk of primary outcomes in the study group remained consistent regardless of the episode of COVID-19 (primary infection: HR, 0.70 [0.64–0.77]; reinfection: HR, 0.81 [0.71–0.93]) and the type of antiviral agents used (NMV-r: HR, 0.77 [0.71–0.84]; MOV: HR, 0.69 [0.59–0.82]). However, although the lower risk of primary outcomes in the study group did not change according to the status of booster vaccination, statistical significance was only observed for those without booster vaccine (HR, 0.74 [0.68–0.80]), and not for those receiving booster vaccine (HR, 0.81 [0.55–1.20]) (Fig. 4).

## Discussion

This first real-world study aimed to assess the effectiveness of novel oral antiviral agents in managing COVID-19 among high-risk patients during the Omicron JN.1 subvariant wave. Through PSM and analysis of data from the TriNetX platform, we identified significant reductions in short-term adverse outcomes among patients receiving oral antiviral treatment compared to those not receiving antiviral agents. Specifically, oral antiviral agents were associated with a significant 23% reduction in the risk of all-cause ED visits, hospitalization, or mortality. Furthermore, they were associated with a 22% reduction in the risk of ED visits, a 14% reduction in the risk of hospitalization, and an impressive 83% reduction in the risk of death. These findings remained consistent across most subgroup analyses, irrespective of age, sex, underlying disease, the episode of infection, and the type of antiviral agents used. Such consistency underscores the robustness of the observed benefits of oral antiviral therapy across diverse patient populations. These results align with previous studies demonstrating the efficacy of oral antiviral agents, such as NMV-r or MOV, in reducing the severity and duration of illness in COVID-19 patients infected with other SARS-CoV-2 variants prior to the Omicron JN.1 subvariant wave [29–39]. The study's findings add to the growing body of evidence supporting the potential benefits of oral antiviral agents in treating COVID-19. They underscore the importance of early intervention with oral antiviral therapy, particularly in high-risk patient populations. By reducing the risk of severe outcomes, including hospitalization and death, oral antiviral agents offer a promising therapeutic option for managing COVID-19 and mitigating its impact on healthcare systems and public health.

Despite consistent benefits observed across almost all subgroup analyses, indicating the potential effectiveness of oral antiviral agents across diverse patient populations, there was a notable difference in the subgroup analysis of individuals with and without booster vaccination. While significant benefits of oral antiviral treatment were evident in individuals without booster vaccination, there was a lack of significant benefit observed in those who had received booster vaccination. This finding aligns with previous research suggesting that effective vaccination can prevent the progression of COVID-19 and may attenuate the effects of antiviral treatment [39, 40]. However, it is worth noting that only a small proportion of patients in our study population had received booster vaccination, constituting less than 2% of the total before PSM. Consequently, oral antiviral agents remain an effective therapeutic option for the vast majority of patients included in this real-world study. Nonetheless, further investigation is warranted to elucidate the underlying mechanisms behind this observation and to comprehensively evaluate the impact of booster vaccination on the outcomes of antiviral treatment. it is crucial to recognize that only a small fraction of patients in our study population had received booster vaccination, comprising less than 2% of the total before PSM. As a result, oral antiviral agents retain their status as an effective therapeutic choice for the overwhelming majority of patients enrolled in this real-world study. However, there is a pressing need for further investigation to uncover the underlying mechanisms driving this observation and to conduct a comprehensive assessment of the impact of booster vaccination on the outcomes of antiviral treatment. In the meanwhile, healthcare authorities should actively seek to understand the reasons behind vaccine hesitancy and fatigue and develop effective strategies to enhance the uptake of booster vaccines. By promoting vaccination uptake and ensuring widespread coverage of booster doses, healthcare systems can strengthen their response to the ongoing COVID-19 pandemic and reduce the burden of disease on individuals and communities.

The findings of this real-world study also revealed that less than one-third of individuals with an elevated risk for disease progression received antiviral agents, suggesting that a substantial proportion of patients who could potentially benefit from antiviral treatment did not receive these effective interventions. Several factors may contribute to the underutilization of antiviral therapy in this population. First, concerns about the risk of drug-related adverse effects may deter healthcare providers from prescribing antiviral agents, particularly in patients with underlying comorbidities. Second, healthcare providers may be concerned about potential drug-drug interactions between antiviral agents and other medications commonly used to manage comorbidities in high-risk patients. Lastly, poor accessibility to antiviral medications may present a barrier to treatment initiation for high-risk COVID-19 patients. However, addressing these barriers to antiviral therapy is paramount to ensure equitable access to effective treatments for high-risk COVID-19 patients. Healthcare systems must prioritize efforts to educate providers about the benefits and safety profile of antiviral agents, enhance surveillance and management of potential drug interactions, and improve the availability and accessibility of antiviral medications. Additionally, strategies to mitigate barriers related to cost, distribution, and healthcare access are essential to ensure that all eligible patients have the opportunity to receive timely and appropriate antiviral treatment. By addressing these challenges, healthcare providers can optimize the management of high-risk COVID-19 patients and reduce the burden of severe illness and mortality.

This study had several strengths. By utilizing epidemiological data from the United States CDC and the US Collaborative network through the TriNetX platform, we were able to identify the cohort particularly affected by the Omicron JN.1 epidemic. Leveraging a large, multi-institutional database allowed us to capture a substantial and diverse patient population involved in the Omicron JN.1 wave. The inclusion of comprehensive patient data, such as demographic characteristics, comorbidities, and treatment information, enabled detailed subgroup analyses and enhanced the generalizability of our findings. PSM was used to minimize confounding factors, aligning our study with Target Trial Emulation principles. Given this robust design, we believe that our analysis of the Omicron JN.1 wave in the US could serve as a valuable resource for the global response to the Omicron JN.1 wave.

However, several limitations should be acknowledged. First, as with any retrospective study, selection bias and unmeasured confounding cannot be entirely ruled out. While PSM was applied to balance baseline characteristics, residual confounding may persist, particularly due to the lack of detailed severity metrics such as the WHO severity score and Charlson Comorbidity Index. However, to minimize this issue, we included only outpatient antivirals per IDSA guidelines for mild-to-moderate disease and excluded patients requiring hospital-level care. Second, requiring at least three medical visits within three months may have favored patients with higher health-seeking behavior or greater disease severity, potentially limiting generalizability. However, this criterion was necessary to ensure regular follow-up, minimize loss to follow-up, and maintain comprehensive case capture for outcome analysis. Despite this, some patients may have sought care outside the TriNetX network, leading to potential underestimation of certain events. This is an inherent limitation of database studies. Third, reliance on electronic health record data may introduce errors or inconsistencies in coding and documentation, affecting the accuracy of outcome ascertainment. For example, patients in the control group may have received antiviral treatment outside the network, which was not captured in our dataset, potentially leading to misclassification bias. The TriNetX database also does not provide disease-specific or COVID-19-related emergency department visits, hospitalizations, or mortality, which may result in overestimation or underestimation of these outcomes. Further real-world studies specifically evaluating COVID-19-related emergency visits, hospitalizations, and mortality are needed. Additionally, the definition of the index date may introduce potential immortal time bias, as patients in the study group must survive until antiviral initiation. However, since antiviral treatment was required to be initiated within 5 days of symptom onset, the window for immortal time was minimal. To further address this concern, we conducted a sensitivity analysis excluding patients who died within 5 days of COVID-19 diagnosis, and the results aligned with our study outcomes (Supplemental Table 5). Furthermore, the small sample size of the booster-vaccinated subgroup limited statistical power, making it difficult to detect a significant effect. The observed lack of benefit in this group may be due to low event rates rather than an actual absence of efficacy. Besides, the observational nature of the study precludes causal inference, and further randomized controlled trials are needed to confirm the efficacy of oral antiviral agents in managing COVID-19. Lastly, due to epidemiological constraints, this study focused exclusively on the Omicron JN.1 wave in the US, where government policies, public health strategies, and patient characteristics might differ from those elsewhere in the world. Specifically, only FDA-approved oral antivirals were considered, excluding novel agents such as ensitrelvir and other emerging treatments approved elsewhere but not yet in the U.S. This may limit the generalizability of our findings to settings where these newer treatments are available.

In conclusion, our study demonstrated the effectiveness of novel oral antiviral agents in mitigating the progression of COVID-19 among high-risk patients during the Omicron JN.1 subvariant wave. This real-world study provides updated and robust evidence supporting the effectiveness of oral antiviral agents in managing COVID-19 among high-risk patients during the Omicron JN.1 subvariant wave. The significant reductions in adverse outcomes observed underscore the importance of early intervention with oral antiviral therapy, particularly in vulnerable populations. However, the lack of significant benefit observed in individuals with booster vaccination highlights the need for further research to elucidate the impact of vaccination status on treatment outcomes. Healthcare systems must prioritize efforts to address barriers to antiviral therapy utilization and promote widespread coverage of booster vaccination to optimize the management of high-risk COVID-19 patients and reduce the burden of severe illness and mortality.

## Supplementary Information

Supplementary Material 1.

## Data Availability

All data generated or analyzed during this study are included in this published article and will be available upon request to CCL.
