# Supplementary material for: Clinical effectiveness of oral antiviral treatment for non-hospitalized high-risk patients with COVID-19 during Omicron JN.1 subvariant wave: a US-based propensity-matched cohort study
Source: Pneumonia (Nathan). 2025 May 25;17:12. doi: 10.1186/s41479-025-00168-w (PMC12103777; doi:10.1186/s41479-025-00168-w)
Supplement: Supplementary file 1 — Supplementary Material 1. [file 41479_2025_168_MOESM1_ESM.pdf]

Supplemental Table 1. Demographic, diagnostic, procedural, medication, visit, and laboratory codes utilized in the definition of the cohorts.

Category	Code	Description
**#1 Age at least 18 years**		
demographics	Age	Age (at least 18 years)
**#2 Visited HCOs at least 3 times between Feb 1, 2024 and Apr 30, 2024**		
(must have any of the following between Feb 1, 2024 and Apr 30, 2024)		
visit	TNX:Visit	Number of occurrences: greater than or equal to 3 instances
**#3 Diagnosed with COVID-19 between Feb 1, 2024 and Apr 30, 2024**		
(must have any of the following between Feb 1, 2024 and Apr 30, 2024)		
laboratory	TNX:9088	SARS coronavirus 2 and related RNA [Presence] (labResult: Positive)
diagnosis	UMLS:ICD10CM:U07.1	COVID-19
diagnosis	UMLS:ICD10CM:U07.2	COVID-19, virus not identified (WHO)
diagnosis	UMLS:ICD10CM:J12.81	Pneumonia due to SARS-associated coronavirus
diagnosis	UMLS:ICD10CM:J12.82	Pneumonia due to coronavirus disease 2019
diagnosis	UMLS:ICD10CM:B34.2	Coronavirus infection, unspecified
diagnosis	UMLS:ICD10CM:B97.2	Coronavirus as the cause of diseases classified elsewhere
diagnosis	UMLS:ICD10CM:B97.21	SARS-associated coronavirus as the cause of diseases classified elsewhere
diagnosis	UMLS:ICD10CM:B97.29	Other coronavirus as the cause of diseases classified elsewhere
laboratory	UMLS:LNC:94309-2	SARS-CoV-2 (COVID-19) RNA [Presence] in Specimen by NAA with probe detection (labResult: Positive)
laboratory	UMLS:LNC:94500-6	SARS-CoV-2 (COVID-19) RNA [Presence] in Respiratory specimen by NAA with probe detection (labResult: Positive)
laboratory	UMLS:LNC:94502-2	SARS-related coronavirus RNA [Presence] in Respiratory specimen by NAA with probe detection (labResult: Positive)
laboratory	UMLS:LNC:94558-4	SARS-CoV-2 (COVID-19) Ag [Presence] in Respiratory specimen by Rapid immunoassay (labResult: Positive)
laboratory	UMLS:LNC:94565-9	SARS-CoV-2 (COVID-19) RNA [Presence] in Nasopharynx by NAA with non-probe detection (labResult: Positive)
laboratory	UMLS:LNC:94759-8	SARS-CoV-2 (COVID-19) RNA [Presence] in Nasopharynx by NAA with probe detection (labResult: Positive)
laboratory	UMLS:LNC:94845-5	SARS-CoV-2 (COVID-19) RNA [Presence] in Saliva (oral fluid) by NAA with probe detection (labResult: Positive)
laboratory	UMLS:LNC:95406-5	SARS-CoV-2 (COVID-19) RNA [Presence] in Nose by NAA with probe detection (labResult: Positive)

laboratory	UMLS:LNC:95608-6	SARS-CoV-2 (COVID-19) RNA [Presence] in Respiratory specimen by NAA with non-probe detection (labResult: Positive)
laboratory	UMLS:LNC:96119-3	SARS-CoV-2 (COVID-19) Ag [Presence] in Upper respiratory specimen by Immunoassay (labResult: Positive)

#4 High risk comorbid conditions for COVID-19 progression

(must have any of the following comorbid condition occurred within 1 year and 1 month before the diagnosis using #3)

diagnosis	UMLS:ICD10CM:D49-D49	Neoplasms of unspecified behavior (D49)
diagnosis	UMLS:ICD10CM:D37-D48	Neoplasms of uncertain behavior, polycythemia vera and myelodysplastic syndromes
diagnosis	UMLS:ICD10CM:C7A-C7A	Malignant neuroendocrine tumors (C7A)
diagnosis	UMLS:ICD10CM:C81-C96	Malignant neoplasms of lymphoid, hematopoietic and related tissue
diagnosis	UMLS:ICD10CM:C7B-C7B	Secondary neuroendocrine tumors (C7B)
diagnosis	UMLS:ICD10CM:C76-C80	Malignant neoplasms of ill-defined, other secondary and unspecified sites
diagnosis	UMLS:ICD10CM:C73-C75	Malignant neoplasms of thyroid and other endocrine glands
diagnosis	UMLS:ICD10CM:C69-C72	Malignant neoplasms of eye, brain and other parts of central nervous system
diagnosis	UMLS:ICD10CM:C64-C68	Malignant neoplasms of urinary tract
diagnosis	UMLS:ICD10CM:C60-C63	Malignant neoplasms of male genital organs
diagnosis	UMLS:ICD10CM:C51-C58	Malignant neoplasms of female genital organs
diagnosis	UMLS:ICD10CM:C50-C50	Malignant neoplasms of breast (C50)
diagnosis	UMLS:ICD10CM:C45-C49	Malignant neoplasms of mesothelial and soft tissue
diagnosis	UMLS:ICD10CM:C43-C44	Melanoma and other malignant neoplasms of skin
diagnosis	UMLS:ICD10CM:C40-C41	Malignant neoplasms of bone and articular cartilage
diagnosis	UMLS:ICD10CM:C30-C39	Malignant neoplasms of respiratory and intrathoracic organs
diagnosis	UMLS:ICD10CM:C15-C26	Malignant neoplasms of digestive organs
diagnosis	UMLS:ICD10CM:C00-C14	Malignant neoplasms of lip, oral cavity and pharynx
diagnosis	UMLS:ICD10CM:D80-D89	Certain disorders involving the immune mechanism
diagnosis	UMLS:ICD10CM:N18	Chronic kidney disease (CKD)
diagnosis	UMLS:ICD10CM:K72.9	Hepatic failure, unspecified
diagnosis	UMLS:ICD10CM:K72.1	Chronic hepatic failure
diagnosis	UMLS:ICD10CM:K71.9	Toxic liver disease, unspecified
diagnosis	UMLS:ICD10CM:K71.8	Toxic liver disease with other disorders of liver
diagnosis	UMLS:ICD10CM:K71.6	Toxic liver disease with hepatitis, not elsewhere classified
diagnosis	UMLS:ICD10CM:K71.5	Toxic liver disease with chronic active hepatitis
diagnosis	UMLS:ICD10CM:K71.4	Toxic liver disease with chronic lobular hepatitis
diagnosis	UMLS:ICD10CM:K71.3	Toxic liver disease with chronic persistent hepatitis
diagnosis	UMLS:ICD10CM:K71.1	Toxic liver disease with hepatic necrosis
diagnosis	UMLS:ICD10CM:K71.0	Toxic liver disease with cholestasis
diagnosis	UMLS:ICD10CM:K77	Liver disorders in diseases classified elsewhere

diagnosis	UMLS:ICD10CM:K75	Other inflammatory liver diseases
diagnosis	UMLS:ICD10CM:K74	Fibrosis and cirrhosis of liver
diagnosis	UMLS:ICD10CM:I20-I25	Ischemic heart diseases
diagnosis	UMLS:ICD10CM:I50	Heart failure
diagnosis	UMLS:ICD10CM:K73	Chronic hepatitis, not elsewhere classified
diagnosis	UMLS:ICD10CM:I60-I69	Cerebrovascular diseases
diagnosis	UMLS:ICD10CM:E11	Type 2 diabetes mellitus
diagnosis	UMLS:ICD10CM:E65-E68	Overweight, obesity and other hyperalimentation
diagnosis	UMLS:ICD10CM:K70	Alcoholic liver disease
diagnosis	UMLS:ICD10CM:K71.7	Toxic liver disease with fibrosis and cirrhosis of liver
diagnosis	UMLS:ICD10CM:F01-F99	Mental, Behavioral and Neurodevelopmental disorders
diagnosis	UMLS:ICD10CM:G30	Alzheimer's disease
diagnosis	UMLS:ICD10CM:F10-F19	Mental and behavioral disorders due to psychoactive substance use
diagnosis	UMLS:ICD10CM:F20-F29	Schizophrenia, schizotypal, delusional, and other non-mood psychotic disorders
diagnosis	UMLS:ICD10CM:F30-F39	Mood [affective] disorders
diagnosis	UMLS:ICD10CM:J40-J4A	Chronic lower respiratory diseases
diagnosis	UMLS:ICD10CM:I10-I1A	Hypertensive diseases

#5 Not hospitalized 2 days before to 5 days after the diagnosis

(cannot have any of the following 2 days before to 5 days after the diagnosis using #3)

visit	UMLS:HL7V3.0:VisitType:ACUTE	Visit: Inpatient Acute
visit	UMLS:HL7V3.0:VisitType:IMP	Visit: Inpatient Encounter
visit	UMLS:HL7V3.0:VisitType:NONAC	Visit: Inpatient Non-acute
visit	UMLS:HL7V3.0:VisitType:SS	Visit: Short Stay

#5 Not deceased on the day of diagnosis

(cannot have the following on the day of diagnosis using #3)

demographics	Deceased	Deceased

#6 Use of anti-COVID-19 agents

(Study group: must have any of #6.1 or #6.2 and must not have #6.3 within 5 days of diagnosis; control goup: must not have any of #6.1-3)

#6.1 NMV-r (must have both the following)

medication	NLM:RXNORM:2587892	Nirmatrelvir
medication	NLM:RXNORM:85762	Ritonavir

#6.2 Molnupiravir (must have the following)

medication	NLM:RXNORM:2587901	Molnupiravir

#6.3 Other anti-COVID-19 agents

medication	NLM:RXNORM:2284718	Remdesivir
medication	NLM:RXNORM:2587300	Tixagevimab
medication	NLM:RXNORM:2587306	Cilgavimab
medication	NLM:RXNORM:2592360	Bebtelovimab

procedure	UMLS:ICD10PCS:XW14325	Transfusion of Convalescent Plasma (Nonautologous) into Central Vein, Percutaneous Approach, New Technology Group 5
procedure	UMLS:ICD10PCS:XW13325	Transfusion of Convalescent Plasma (Nonautologous) into Peripheral Vein, Percutaneous Approach, New Technology Group 5

Supplemental Table 2. Demographic, diagnostic, and laboratory codes utilized in the definition of covariates.

Codes	Description
AI	Age at Index
F	Female
56370	Black or African American
M	Male
75301	White
1002-5	American Indian or Alaska Native
UNK	Unknown Race
64498	Native Hawaiian or Other Pacific Islander
UN	Unknown Ethnicity
104582	Not Hispanic or Latino
85865	Hispanic or Latino
84373	Other Race
46997	Asian
C00-D49	Neoplasms
F20-F29	Schizophrenia, schizotypal, delusional, and other non-mood psychotic disorders
F30-F39	Mood [affective] disorders
F40-F48	Anxiety, dissociative, stress-related, somatoform and other nonpsychotic mental disorders
F50-F59	Behavioral syndromes associated with physiological disturbances and physical factors
F70-F79	Intellectual Disabilities
F17	Nicotine dependence
Z87.891	Personal history of nicotine dependence
D80-D89	Certain disorders involving the immune mechanism
Z21	Asymptomatic human immunodeficiency virus [HIV] infection status
B20	Human immunodeficiency virus [HIV] disease
N18	Chronic kidney disease (CKD)
J40-J4A	Chronic lower respiratory diseases
E84	Cystic fibrosis
K70-K77	Diseases of liver
E66	Overweight and obesity
E08-E13	Diabetes mellitus
I10-I1A	Hypertensive diseases
I20-I25	Ischemic heart diseases
I26-I28	Pulmonary heart disease and diseases of pulmonary circulation
I30-I52	Other forms of heart disease (deprecated 2021)
I30-I5A	Other forms of heart disease
I60-I69	Cerebrovascular diseases
Z59	Problems related to housing and economic circumstances
9083	BMI

	25 - 30 kg/m2
	≥ 30 kg/m2

Supplemental Table 3. Diagnostic, visit, and procedural codes utilized in the definition of outcomes. Index date: the first date of antiviral agent prescription for the study group, the first date of diagnosis for control group

Outcome time window: 3 days to 1 month after the index date

Category	Code	Description
**#1 Composite outcomes (must have #1.1 or #1.2 or #1.3)**		
**#1.1: All-cause ED visit (must have any of the following)**		
visit	UMLS:HL7V3.0:VisitType:EMER	Visit: Emergency
**#1.2: All-cause hospitalization (must have any of the following)**		
visit	UMLS:HL7V3.0:VisitType:ACUTE	Visit: Inpatient Acute
visit	UMLS:HL7V3.0:VisitType:IMP	Visit: Inpatient Encounter
visit	UMLS:HL7V3.0:VisitType:NONAC	Visit: Inpatient Non-acute
visit	UMLS:HL7V3.0:VisitType:SS	Visit: Short Stay
**#1.3 All-cause mortality (must have the following)**		
Demographics	Deceased	Deceased

Supplemental Table 4. Demographic, diagnostic, procedural, and medication codes utilized in the definition of the subgroup analysis

**#1 Age**		
**#1.1 Age <65**		
demographics	Age	between 18 and 64 years
**#1.2 Age ≥ 65**		
demographics	Age	≥ 65 years
**#2 Sex**		
**#2.1 Female**		
demographics	UMLS:HL7V3.0:Gender:F	Female
**#2.2 Male**		
demographics	UMLS:HL7V3.0:Gender:M	Male
**#3Comorbid conditions** (conditions occurred within 1 year and 1 month before the index date)		
**#3.1: Cardiovascular diseases**		
diagnosis	UMLS:ICD10CM:I10-I15	Hypertensive diseases (deprecated 2018)
diagnosis	UMLS:ICD10CM:I10-I1A	Hypertensive diseases
diagnosis	UMLS:ICD10CM:I20-I25	Ischemic heart diseases
diagnosis	UMLS:ICD10CM:I26-I28	Pulmonary heart disease and diseases of pulmonary circulation
diagnosis	UMLS:ICD10CM:I30-I52	Other forms of heart disease (deprecated 2021)
diagnosis	UMLS:ICD10CM:I30-I5A	Other forms of heart disease
**#3.2: Cerebrovascular diseases**		
diagnosis	UMLS:ICD10CM:I60-I69	Cerebrovascular diseases
**#3.3: Chronic liver diseases**		
diagnosis	UMLS:ICD10CM:K72.9	Hepatic failure, unspecified
diagnosis	UMLS:ICD10CM:K72.1	Chronic hepatic failure
diagnosis	UMLS:ICD10CM:K71.9	Toxic liver disease, unspecified
diagnosis	UMLS:ICD10CM:K71.8	Toxic liver disease with other disorders of liver
diagnosis	UMLS:ICD10CM:K71.6	Toxic liver disease with hepatitis, not elsewhere classified
diagnosis	UMLS:ICD10CM:K71.5	Toxic liver disease with chronic active hepatitis
diagnosis	UMLS:ICD10CM:K71.4	Toxic liver disease with chronic lobular hepatitis
diagnosis	UMLS:ICD10CM:K71.3	Toxic liver disease with chronic persistent hepatitis
diagnosis	UMLS:ICD10CM:K71.1	Toxic liver disease with hepatic necrosis
diagnosis	UMLS:ICD10CM:K71.0	Toxic liver disease with cholestasis
diagnosis	UMLS:ICD10CM:K77	Liver disorders in diseases classified elsewhere
diagnosis	UMLS:ICD10CM:K75	Other inflammatory liver diseases
diagnosis	UMLS:ICD10CM:K74	Fibrosis and cirrhosis of liver
diagnosis	UMLS:ICD10CM:K73	Chronic hepatitis, not elsewhere classified
diagnosis	UMLS:ICD10CM:K70	Alcoholic liver disease
diagnosis	UMLS:ICD10CM:K71.7	Toxic liver disease with fibrosis and cirrhosis of liver
**#3.4: Chronic lower lung diseases**		

diagnosis	UMLS:ICD10CM:J40-J47	Chronic lower respiratory diseases

#3.5: Chronic kidney diseases

diagnosis	UMLS:ICD10CM:N18	Chronic kidney disease (CKD)

#3.6: Type 2 diabetes mellitus

diagnosis	UMLS:ICD10CM:E11	Type 2 diabetes mellitus

#3.7: Overweight and obesity

diagnosis	UMLS:ICD10CM:E65-E68	Overweight, obesity and other hyperalimentation
laboratory	9083	BMI ≥ 25 kg/m2

#4 SARS-CoV-2 infection status

#4.1 Primary infection (must not have the following 3 months prior to the index date)

laboratory	TNX:9088	SARS coronavirus 2 and related RNA [Presence] (labResult: Positive)
diagnosis	UMLS:ICD10CM:U07.1	COVID-19
diagnosis	UMLS:ICD10CM:U07.2	COVID-19, virus not identified (WHO)
diagnosis	UMLS:ICD10CM:J12.81	Pneumonia due to SARS-associated coronavirus
diagnosis	UMLS:ICD10CM:J12.82	Pneumonia due to coronavirus disease 2019
diagnosis	UMLS:ICD10CM:B34.2	Coronavirus infection, unspecified
diagnosis	UMLS:ICD10CM:B97.2	Coronavirus as the cause of diseases classified elsewhere
diagnosis	UMLS:ICD10CM:B97.21	SARS-associated coronavirus as the cause of diseases classified elsewhere
diagnosis	UMLS:ICD10CM:B97.29	Other coronavirus as the cause of diseases classified elsewhere
laboratory	UMLS:LNC:94309-2	SARS-CoV-2 (COVID-19) RNA [Presence] in Specimen by NAA with probe detection (labResult: Positive)
laboratory	UMLS:LNC:94500-6	SARS-CoV-2 (COVID-19) RNA [Presence] in Respiratory specimen by NAA with probe detection (labResult: Positive)
laboratory	UMLS:LNC:94502-2	SARS-related coronavirus RNA [Presence] in Respiratory specimen by NAA with probe detection (labResult: Positive)
laboratory	UMLS:LNC:94558-4	SARS-CoV-2 (COVID-19) Ag [Presence] in Respiratory specimen by Rapid immunoassay (labResult: Positive)
laboratory	UMLS:LNC:94565-9	SARS-CoV-2 (COVID-19) RNA [Presence] in Nasopharynx by NAA with non-probe detection (labResult: Positive)
laboratory	UMLS:LNC:94759-8	SARS-CoV-2 (COVID-19) RNA [Presence] in Nasopharynx by NAA with probe detection (labResult: Positive)
laboratory	UMLS:LNC:94845-5	SARS-CoV-2 (COVID-19) RNA [Presence] in Saliva (oral fluid) by NAA with probe detection (labResult: Positive)
laboratory	UMLS:LNC:95406-5	SARS-CoV-2 (COVID-19) RNA [Presence] in Nose by NAA with probe detection (labResult: Positive)
laboratory	UMLS:LNC:95608-6	SARS-CoV-2 (COVID-19) RNA [Presence] in Respiratory specimen by NAA with non-probe detection (labResult: Positive)

laboratory	UMLS:LNC:96119-3	SARS-CoV-2 (COVID-19) Ag [Presence] in Upper respiratory specimen by Immunoassay (labResult: Positive)

#4.2 Reinfection (must have any of #4.1 3 months prior to the index date)

#5 Outpatient COVID-19 antiviral treatment

#5.1 Treatment with NMV-r (must have the following within 5 days of diagnosis)

medication	NLM:RXNORM:2587892	nirmatrelvir
medication	NLM:RXNORM:85762	ritonavir

#5.2 Treatment with Molnupiravir

medication	NLM:RXNORM:2587901	molnupiravir

#6 COVID-19 booster vaccination status (excluded those who received booster vaccines within 2 weeks prior to the index date)

#5.1 No (must not have any of following within 6 months prior to the index date)

procedure	UMLS:CPT:91300	Severe acute respiratory syndrome coronavirus 2 (SARS-CoV-2) (coronavirus disease [COVID-19]) vaccine, mRNA-LNP, spike protein, preservative free, 30 mcg/0.3 mL dosage, diluent reconstituted, for intramuscular use
procedure	UMLS:CPT:91301	Severe acute respiratory syndrome coronavirus 2 (SARS-CoV-2) (coronavirus disease [COVID-19]) vaccine, mRNA-LNP, spike protein, preservative free, 100 mcg/0.5 mL dosage, for intramuscular use
procedure	UMLS:CPT:91302	Severe acute respiratory syndrome coronavirus 2 (SARS-CoV-2) (coronavirus disease [COVID-19]) vaccine, DNA, spike protein, chimpanzee adenovirus Oxford 1 (ChAdOx1) vector, preservative free, 5x1010 viral particles/0.5 mL dosage, for intramuscular use
procedure	UMLS:CPT:91303	Severe acute respiratory syndrome coronavirus 2 (SARS-CoV-2) (coronavirus disease [COVID-19]) vaccine, DNA, spike protein, adenovirus type 26 (Ad26) vector, preservative free, 5x1010 viral particles/0.5 mL dosage, for intramuscular use
procedure	UMLS:CPT:91304	Severe acute respiratory syndrome coronavirus 2 (SARS-CoV-2) (coronavirus disease [COVID-19]) vaccine, recombinant spike protein nanoparticle, saponin-based adjuvant, preservative free, 5 mcg/0.5 mL dosage, for intramuscular use
procedure	UMLS:CPT:91305	Severe acute respiratory syndrome coronavirus 2 (SARS-CoV-2) (coronavirus disease [COVID-19]) vaccine, mRNA-LNP, spike protein, preservative free, 30 mcg/0.3 mL dosage, tris-sucrose formulation, for intramuscular use
procedure	UMLS:CPT:91306	Severe acute respiratory syndrome coronavirus 2 (SARS-CoV-2) (coronavirus disease [COVID-19]) vaccine, mRNA-LNP, spike protein, preservative free, 50 mcg/0.25 mL dosage, for intramuscular use
procedure	UMLS:CPT:91307	Severe acute respiratory syndrome coronavirus 2 (SARS-CoV-2) (coronavirus disease [COVID-19]) vaccine, mRNA-LNP, spike protein, preservative free, 10 mcg/0.2 mL dosage, diluent reconstituted, tris-sucrose formulation, for intramuscular use

procedure	UMLS:CPT:91308	Severe acute respiratory syndrome coronavirus 2 (SARS-CoV-2) (coronavirus disease [COVID-19]) vaccine, mRNA-LNP, spike protein, preservative free, 3 mcg/0.2 mL dosage, diluent reconstituted, tris-sucrose formulation, for intramuscular use
procedure	UMLS:CPT:91309	Severe acute respiratory syndrome coronavirus 2 (SARS-CoV-2) (coronavirus disease [COVID-19]) vaccine, mRNA-LNP, spike protein, preservative free, 50 mcg/0.5 mL dosage, for intramuscular use
procedure	UMLS:CPT:91311	Severe acute respiratory syndrome coronavirus 2 (SARS-CoV-2) (coronavirus disease [COVID-19]) vaccine, mRNA-LNP, spike protein, preservative free, 25 mcg/0.25 mL dosage, for intramuscular use
procedure	UMLS:CPT:91312	Severe acute respiratory syndrome coronavirus 2 (SARS-CoV-2) (coronavirus disease [COVID-19]) vaccine, mRNA-LNP, bivalent spike protein, preservative free, 30 mcg/0.3 mL dosage, tris-sucrose formulation, for intramuscular use
procedure	UMLS:CPT:91313	Severe acute respiratory syndrome coronavirus 2 (SARS-CoV-2) (coronavirus disease [COVID-19]) vaccine, mRNA-LNP, spike protein, bivalent, preservative free, 50 mcg/0.5 mL dosage, for intramuscular use
procedure	UMLS:CPT:91314	Severe acute respiratory syndrome coronavirus 2 (SARS-CoV-2) (coronavirus disease [COVID-19]) vaccine, mRNA-LNP, spike protein, bivalent, preservative free, 25 mcg/0.25 mL dosage, for intramuscular use
procedure	UMLS:CPT:91315	Severe acute respiratory syndrome coronavirus 2 (SARS-CoV-2) (coronavirus disease [COVID-19]) vaccine, mRNA-LNP, bivalent spike protein, preservative free, 10 mcg/0.2 mL dosage, diluent reconstituted, tris-sucrose formulation, for intramuscular use
procedure	UMLS:CPT:91316	Severe acute respiratory syndrome coronavirus 2 (SARS-CoV-2) (coronavirus disease [COVID-19]) vaccine, mRNA-LNP, spike protein, bivalent, preservative free, 10 mcg/0.2 mL dosage, for intramuscular use
procedure	UMLS:CPT:91317	Severe acute respiratory syndrome coronavirus 2 (SARS-CoV-2) (coronavirus disease [COVID-19]) vaccine, mRNA-LNP, bivalent spike protein, preservative free, 3 mcg/0.2 mL dosage, diluent reconstituted, tris-sucrose formulation, for intramuscular use
procedure	UMLS:CPT:91318	Severe acute respiratory syndrome coronavirus 2 (SARS-CoV-2) (coronavirus disease [COVID-19]) vaccine, mRNA-LNP, spike protein, 3 mcg/0.2 mL dosage, diluent reconstituted, tris-sucrose formulation, for intramuscular use
procedure	UMLS:CPT:91319	Severe acute respiratory syndrome coronavirus 2 (SARS-CoV-2) (coronavirus disease [COVID-19]) vaccine, mRNA-LNP, spike protein, 10 mcg/0.2 mL dosage, tris-sucrose formulation, for intramuscular use

procedure	UMLS:CPT:91320	Severe acute respiratory syndrome coronavirus 2 (SARS-CoV-2) (coronavirus disease [COVID-19]) vaccine, mRNA-LNP, spike protein, 30 mcg/0.3 mL dosage, tris-sucrose formulation, for intramuscular use
procedure	UMLS:CPT:91321	Severe acute respiratory syndrome coronavirus 2 (SARS-CoV-2) (coronavirus disease [COVID-19]) vaccine, mRNA-LNP, 25 mcg/0.25 mL dosage, for intramuscular use
procedure	UMLS:CPT:91322	Severe acute respiratory syndrome coronavirus 2 (SARS-CoV-2) (coronavirus disease [COVID-19]) vaccine, mRNA-LNP, 50 mcg/0.5 mL dosage, for intramuscular use

#5.2 Yes (must have any of #5.1within 6 months prior to the index date)

Supplemental Table 5. Sensitivity analysis of the impact of early mortality exclusion on outcome comparisons

Sensitivity analysis	No. (%) of patients with outcome		HR (95% CI)	Log-rank test *p*-value
	Study group (n=17,516)	Control group (n=17,516)		
**Primary outcome**				
All-cause ED visit, hospitalization or mortality	1,213 (6.9)	1,546 (8.8)	0.76 (0.71, 0.82)	<0.0001
**Secondary outcomes**				
All-cause ED visit	742 (4.2)	901 (5.1)	0.81 (0.73,0.89)	<0.0001
All-cause hospitalization	582 (3.3)	759 (4.3)	0.75 (0.67,0.84)	<0.0001
All-cause mortality	12 (0.1)	43 (0.2)	0.27 (0.144,0.519)	< 0.0001
